# A battery-less and wireless wearable sensor system for identifying bed and chair exits in a pilot trial in hospitalized older people

**DOI:** 10.1371/journal.pone.0185670

**Published:** 2017-10-09

**Authors:** Roberto L. Shinmoto Torres, Renuka Visvanathan, Derek Abbott, Keith D. Hill, Damith C. Ranasinghe

**Affiliations:** 1 School of Computer Science, University of Adelaide, Adelaide, South Australia, Australia; 2 Auto-ID Lab, University of Adelaide, Adelaide, South Australia, Australia; 3 Adelaide Geriatrics Training and Research with Aged Care Centre, University of Adelaide, Paradise, South Australia, Australia; 4 Aged & Extended Care Services, The Queen Elizabeth Hospital, Central Adelaide Local Health Network, University of Adelaide, Woodville South, South Australia, Australia; 5 Centre for Biomedical Engineering (CBME), University of Adelaide, Adelaide, South Australia, Australia; 6 School of Electrical & Electronic Engineering, University of Adelaide, Adelaide, South Australia, Australia; 7 School of Physiotherapy and Exercise Science, Curtin University, Perth, Western Australia, Australia; National Yang-Ming University, TAIWAN

## Abstract

Falls in hospitals are common, therefore strategies to minimize the impact of these events in older patients and needs to be examined. In this pilot study, we investigate a movement monitoring sensor system for identifying bed and chair exits using a wireless wearable sensor worn by hospitalized older patients. We developed a movement monitoring sensor system that recognizes bed and chair exits. The system consists of a machine learning based activity classifier and a bed and chair exit recognition process based on an activity score function. Twenty-six patients, aged 71 to 93 years old, hospitalized in the Geriatric Evaluation and Management Unit participated in the supervised trials. They wore over their attire a battery-less, lightweight and wireless sensor and performed scripted activities such as getting off the bed and chair. We investigated the system performance in recognizing bed and chair exits in hospital rooms where RFID antennas and readers were in place. The system’s acceptability was measured using two surveys with 0–10 likert scales. The first survey measured the change in user perception of the system before and after a trial; the second survey, conducted only at the end of each trial, measured user acceptance of the system based on a multifactor sensor acceptance model. The performance of the system indicated an overall recall of 81.4%, precision of 66.8% and F-score of 72.4% for joint bed and chair exit recognition. Patients demonstrated improved perception of the system after use with overall score change from 7.8 to 9.0 and high acceptance of the system with score ≥ 6.7 for all acceptance factors. The present pilot study suggests the use of wireless wearable sensors is feasible for detecting bed and chair exits in a hospital environment.

## Introduction

Falls are the leading cause of preventable injuries in hospitalized older people, especially those with dementia or delirium, where 30% of falls result in injury, and 4–6% in serious injury including death [1]. Moreover, about 2.5 million older people are hospitalized annually due to a fall related injury in the U.S. alone [2]. Apart from physical injury and added financial burden from related expenses, falls cause psychological trauma such as fear, loss of confidence, anxiety and depression, which in turn impact on an older person’s independence [3, 4]. In monetary terms, the U.S. Centers for Disease Control and Prevention (CDC) estimates for 2016 the hospital cost of a single fall-related injury at US$35 777 and the direct medical costs for falls injuries to reach US$34.8 billion (both amounts adjusted for inflation since 2013) [5].

Falls are reported to commonly occur in patients’ rooms (84%) including those around the bed and chair [6, 7] or in the toilet (11%); in terms of activities at the time of the fall, most falls occur when ambulating (19%), especially without the necessary walking aid [1]. In addition, risk of falling increases as cognitive functions decline in older people [8, 9]. Best practice standards for falls prevention in hospitals and nursing homes include the use of safe footwear, review of medications or use of bed and chair exit alarm systems for patients at risk of falling to provide timely alerts to staff to lend assistance to patients attempting to ambulate unsupervised [10]. However, falls rates remain high [11–13].

Two recent long-term fall prevention trials using pressure sensors on beds and chairs by Sahota et al. [14] and Shorr et al. [15] have reported no decrease in falls rate; this lack of success can be partially attributed to “alarm fatigue” due to the very low specificity of pressure sensors (about 0.3% in Capezuti et al. [4]) resulting in high number of false alarms. In addition to the poor performance of pressure mats placed over the chair or mattress, these sensors require constant maintenance such as cleaning and disinfection as pressure mats are likely to be in contact with body fluids, and thus increasing the workload of staff. Moreover, audible alarms [15] result in ‘noise’ that distresses patients, especially those with cognitive impairment. Multiple studies have also used video images for preventing falls [16–18]. However, previous research has shown the manifestation of privacy concerns with the use of cameras in older people’s living environment [19]. The study in [19] among community-dwelling older people reports that cameras raised greater privacy concerns than other technologies, even when methods for extracting silhouettes were in place to preserve privacy. Further, the robustness of vision based recognition techniques can be challenged due to multiple reasons such as changing illumination, clutter, dynamic backgrounds, occlusions and, in a hospital context, multiple people, patients or visitors in a single ward room.

Wearable sensors as part of a falls prevention alarm system can provide new opportunities for individualized monitoring of patients [20–22] where human motion data can be collected and transmitted in real time for analysis. More importantly, recent studies [23, 24] suggest that older people have demonstrated interest in small sensors embedded in their clothing. In addition, as sensors are decreasing in size and power consumption, the inclusion of multiple sensors can further extract physiological signals of interest [20–22] along with motion data. Most studies using wearable sensors that target older people have mainly focused on the recognition of activities of daily living [25, 26], gait analysis [27] or the assessment of future falls risk against a validated clinical scale in older people [28–30].

**Fig 1:**
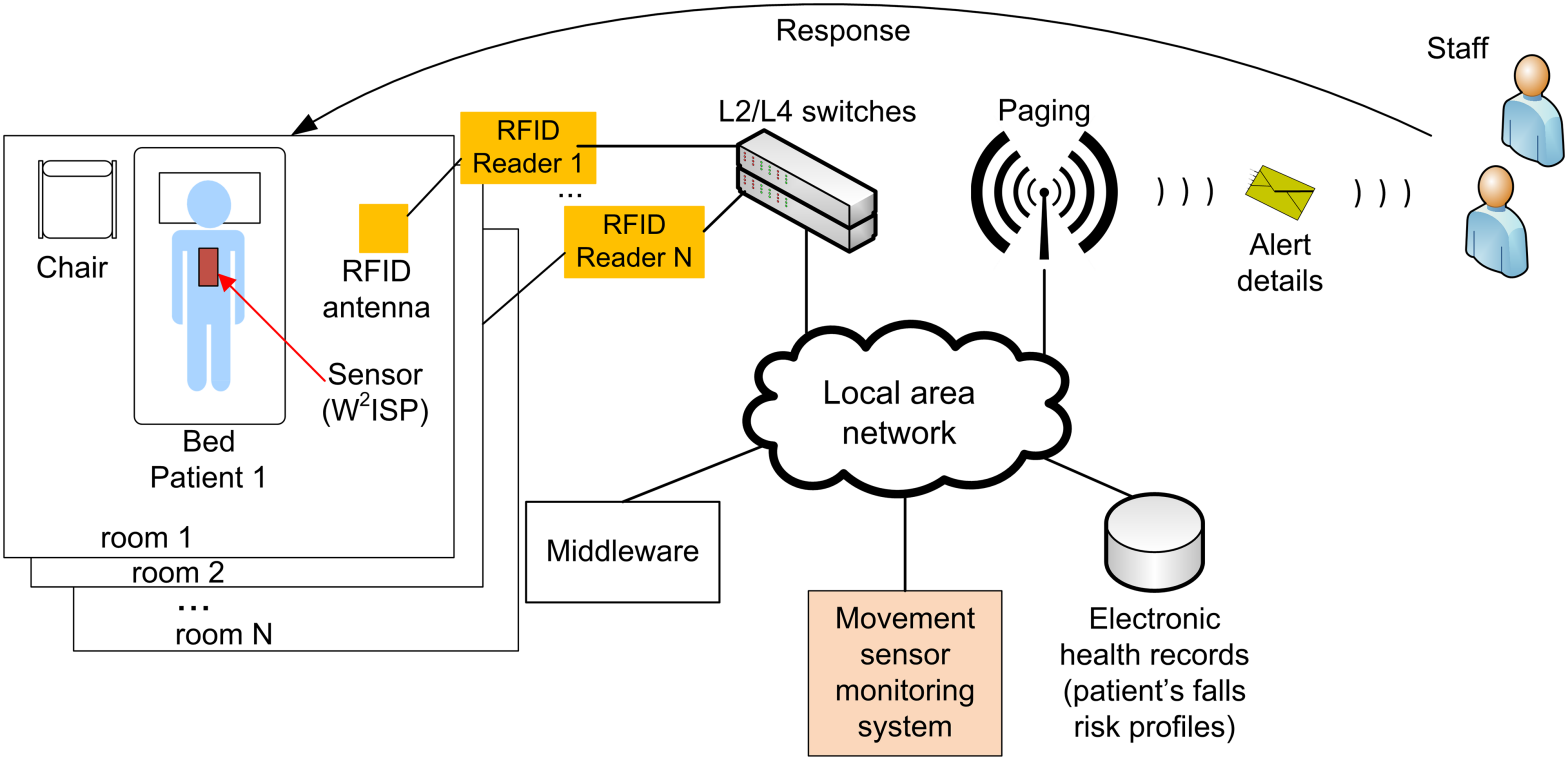
Overview of our technological approach for falls prevention. A patient wears our wearable batteryless sensor, namely a Wearable Wireless Identification and Sensing Platform (W^2^ISP), on top of their clothing. The sensor device sends movement information to the movement monitoring sensor system which issues an alarm to staff when a bed or chair exit is recognized.

Our study, based on the use of a batteryless werarable sensor device, focuses on methods for the recognition of unassisted bed and chair exits as part of a larger intervention strategy for falls prevention as illustrated in Fig 1 [31]. We propose the use of wearable battery-less sensor enabled radio frequency identification (RFID) devices [32], placed on top of patient’s clothes, to collect human motion information [33]. The use of wearable sensors is advantageous as it is low cost, disposable, easy to replace and is maintenance-free. Data from the sensor is collected through the RFID infrastructure (wall or ceiling mounted antennas, and readers) connected to a local area network. The system identifies which patient, via their unique RFID tag identifier, requires clinical supervision to move out of a bed or chair. An alert is issued to staff once an unsupervised bed or chair exit is recognized. Staff responding to the individual patient’s alert may then intervene and prevent a fall from occurring or provide immediate assistance in the event of a fall incident to prevent a ‘long lie’.

We have tested our proposed batteryless sensor device worn on the chest on top of the participant’s clothes in young volunteers [34] and healthy older people [35, 36]. High recognition accuracy of bed exits using a heuristics-based approach in [34] achieved with young people did not perform well in healthy older people. Using machine learning techniques, we were better able to identify bed exits and chair exits in real time [36]. Our studies in healthy younger and older people confirmed the feasibility of using batteryless wearable sensors and the use of a 3 antenna deployment as an effective method to collect motion data from the batteryless wearable sensor. More significantly, our preliminary work has highlighted the need to develop algorithms for the intended target population—i.e. frail hospitalized patients—because of observed differences in posture transitions and ambulation of frail older people and the consequential inability of learned models developed in the domain of healthy older people to adapt effectively to the domain described by frail hospitalized patients. Furthermore, an evaluation of a system’s ability to detect risk movements such as bed and chair exits has not been reported with hospitalized older people, except for the studies by Capezuti et al. [4] where a combination of pressure mats and infrared beams was investigated and Wong et al. [37] which used pressure mats. Most studies to date have focused solely on reporting the occurrence of falls as a metric to determine the efficacy of the tested falls prevention method [13, 38–40]. Unfortunately, the occurrence of falls cannot directly reveal the underlying performance of the tested falls prevention technology; for example other factors such as staffing levels and timeliness of staff to attend to patients can also influence the reported number of falls and thus we cannot determine whether non-prevented falls were caused by failure of the alarming method or other causes. Nevertheless, from the two studies that have measured the performance of the alarming system with hospitalized older people, the study in [4] found that pressure mat based systems generated a large number of falls alarms—a specificity of 0.3% was reported in the study; whereas the system of [37] obtained almost similar false alarms to true alarms, especially at night.

In addition, less than 1.3% of current literature on body worn sensors have considered the perception of end users (e.g. patients) [23] but yet we know that ultimately, the opinion of the end user matters most if the technology is to be successfully translated. Hence, it is also imperative that we investigate the patient’s perception as well as the acceptability of our system. Therefore, the aims of this pilot study are twofold: i) to explore the performance of our wearable sensor based system in identifying the movement transitions of bed and chair exits in hospitalized older people; and ii)to explore the acceptability of the system to inpatients. This is a vital developmental step prior to investigating the system within a randomized control trial (RCT) to confirm cost-effectiveness of the intervention in preventing falls in hospitals.

## Materials and methods

### Ethics statement

This pilot study is designed as a prospective, non-randomized clinical study. The study is approved by the Human Research Ethics Committee of the Queen Elizabeth Hospital (protocol number 2011129) located in Adelaide, South Australia, Australia.

### Technology

#### Wearable sensor technology

**Fig 2:**
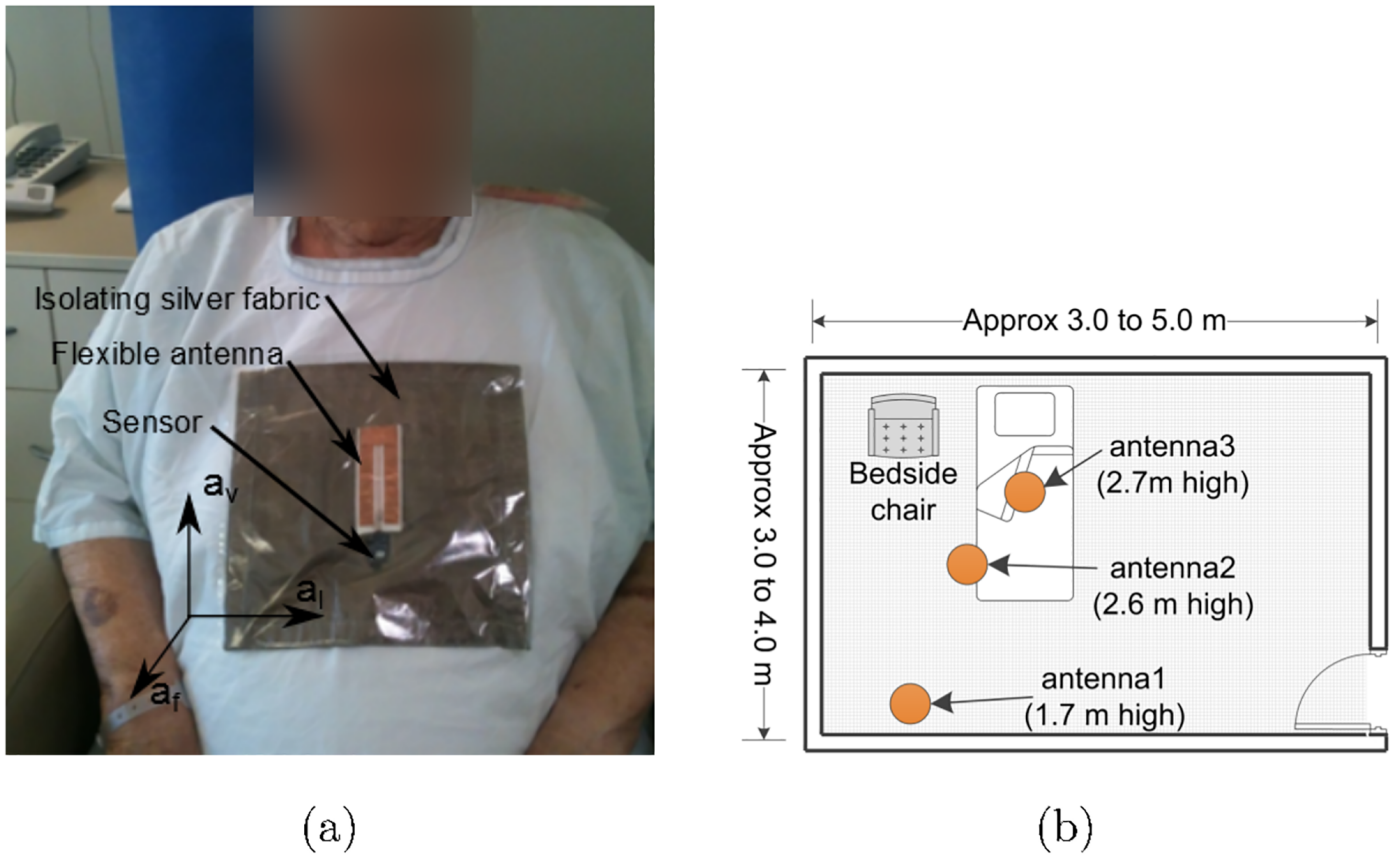
Wireless wearable sensor W^2^ISP and hospital room configuration. (a) Hospital patient wearing a W^2^ISP sensor (≈ 3 g, dimensions: 18 × 20 × 2 mm), flexible antenna (36 × 85 × 2 mm) and isolating silver fabric (230 × 220 mm) on top of a gown for illustrative purposes; also shown are the axes of the accelerometer embedded in the W^2^ISP. (b) Layout of furniture and equipment with antennas shown in circles; antenna2 and antenna3 are at ceiling level on top of bed and antenna1 inclined towards the chair.

**Fig 3:**
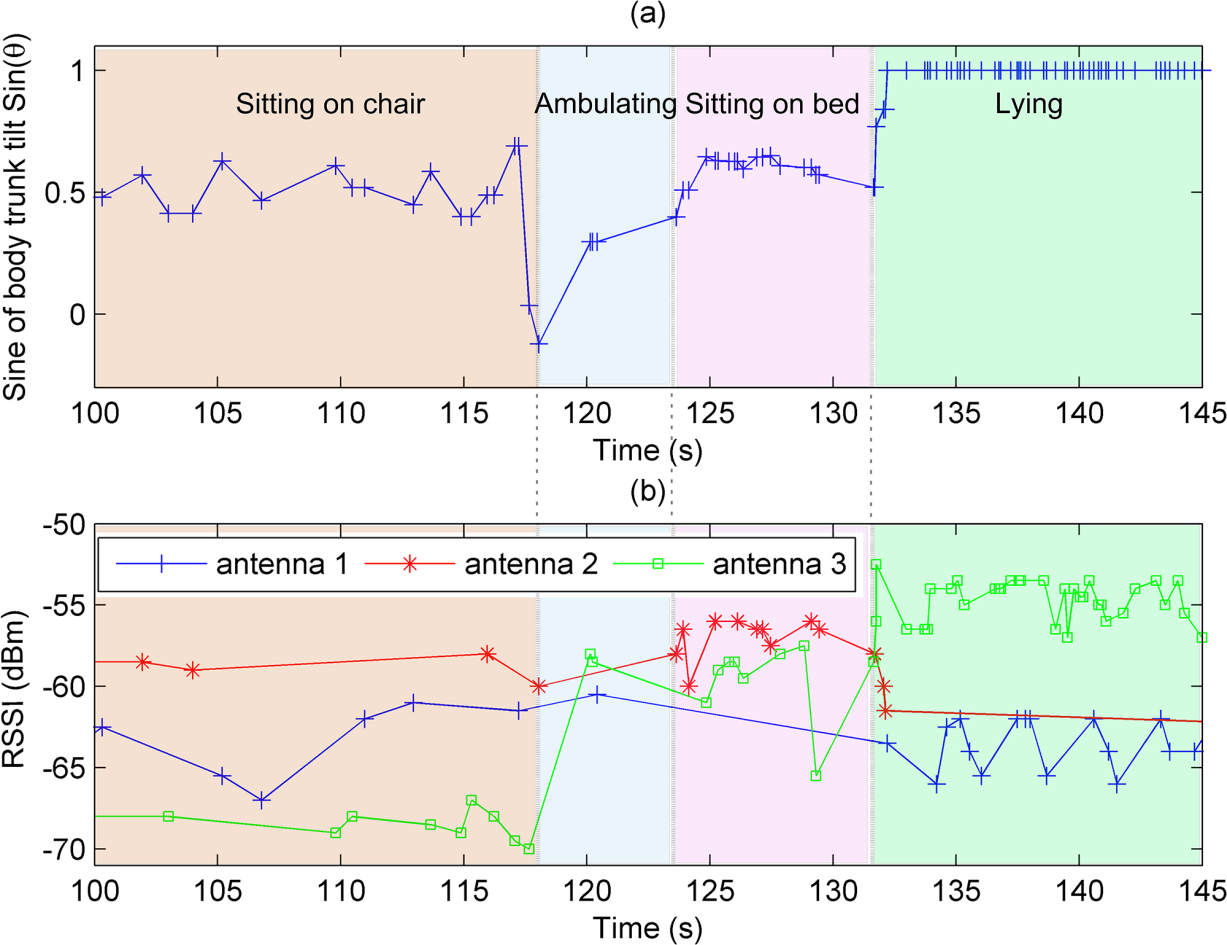
Sensor readings of patient obtained during trial. (a) shows the sine of the body tilting angle (shown in Fig 5(a)) with respect to the vertical. We can also see the sparsity of the sensor readings and the irregular time intervals between sensor readings during activity performance, noted by the varying time intervals between markers on the plot. (b) shows RSSI values for the 3 antennas used in the hospital deployment, we can see the changes in RSSI values across activity boundaries, most notable change is that of antenna 3 (in green) when the patient moves from sitting on chair to ambulating.

We employed a flexible Wearable Wireless Identification and Sensing Platform (W^2^ISP) device developed by our team, suitable for wear over garments at the sternum level as shown in Fig 2(a) [33, 35] (picture obtained with patient’s consent, patient’s face is blurred for anonymization). The W^2^ISP, referred to as simply the *sensor* henceforth, is an RFID tag that integrates a tri-axial accelerometer (ADXL330) and microprocessor (MSP430F2132) with a flexible antenna for patient comfort and a silver fabric to isolate the antenna from the patient; see Fig 2(a). The sensor does not require batteries (i.e. passive device) as it harvests its energy from the electromagnetic (EM) field generated by the RFID reader antennas illuminating it during the interrogation cycles performed by the RFID reader. When adequately powered, the sensor simply backscatters the unique ID and accelerometer information using the incident RF signal from an RFID reader antenna [41]. A consequence of wirelessly powering the sensor with the incident EM fields is that information can only be obtained when the sensor has gathered sufficient power for powering the W^2^ISP circuitry and backscatters data back to the RFID reader. Number of factors, including distance to RFID reader antennas, destructive interference due to multipath, radio frequency band interference and occlusion by RF opaque objects such as the human body can alter the amount of power gathered by our sensor. Hence, we have an irregular reading rate where the time interval between sensor readings varies. In our practical deployment of the sensor, we obtained a sensor read rate of less than 20 reads per second. We can observe the sparse and irregular nature of the sensor data stream from the extract of sensor data recorded from a hospitalized patient shown in Fig 3.

The movement monitoring sensor system collects: i) acceleration readings from the tri-axial accelerometer *a*_*v*_, *a*_*f*_, *a*_*l*_—shown in Fig 2(a) where acceleration components are corrected based on calibration data and converted to *g* values; ii)the measured strength of the received backscattered signal at the RFID reader antenna, called received signal strength indicator (RSSI); iii) RFID reader antenna receiving sensor information; iv) *F* represents frequency values in MHz of the channels used by the RFID reader to query the sensor; and v) RF phase (*ϕ*), the phase angle between the transmitted RF carrier frequency *F* and the backscattered signal from the sensor. The acceleration data captures body trunk movement information related to the activities performed by the participant. RSSI is used as a measure of change in distances of a patient wearing the sensor to the RFID reader antenna (aID) receiving the sensor reading [35]. This is because a sensor located closer to the antenna reports higher RSSI values than a sensor located further away. The phase *ϕ* is used as a source of spatial information (e.g. tag’s velocity and distance) to analyze patient movements [42]. Thus, the collected data for every reading has the information: (*a*_*v*_, *a*_*f*_, *a*_*l*_, RSSI, aID, *F*, *ϕ*). We provide detailed description of the features we have used in Feature extraction section.

**Fig 4:**
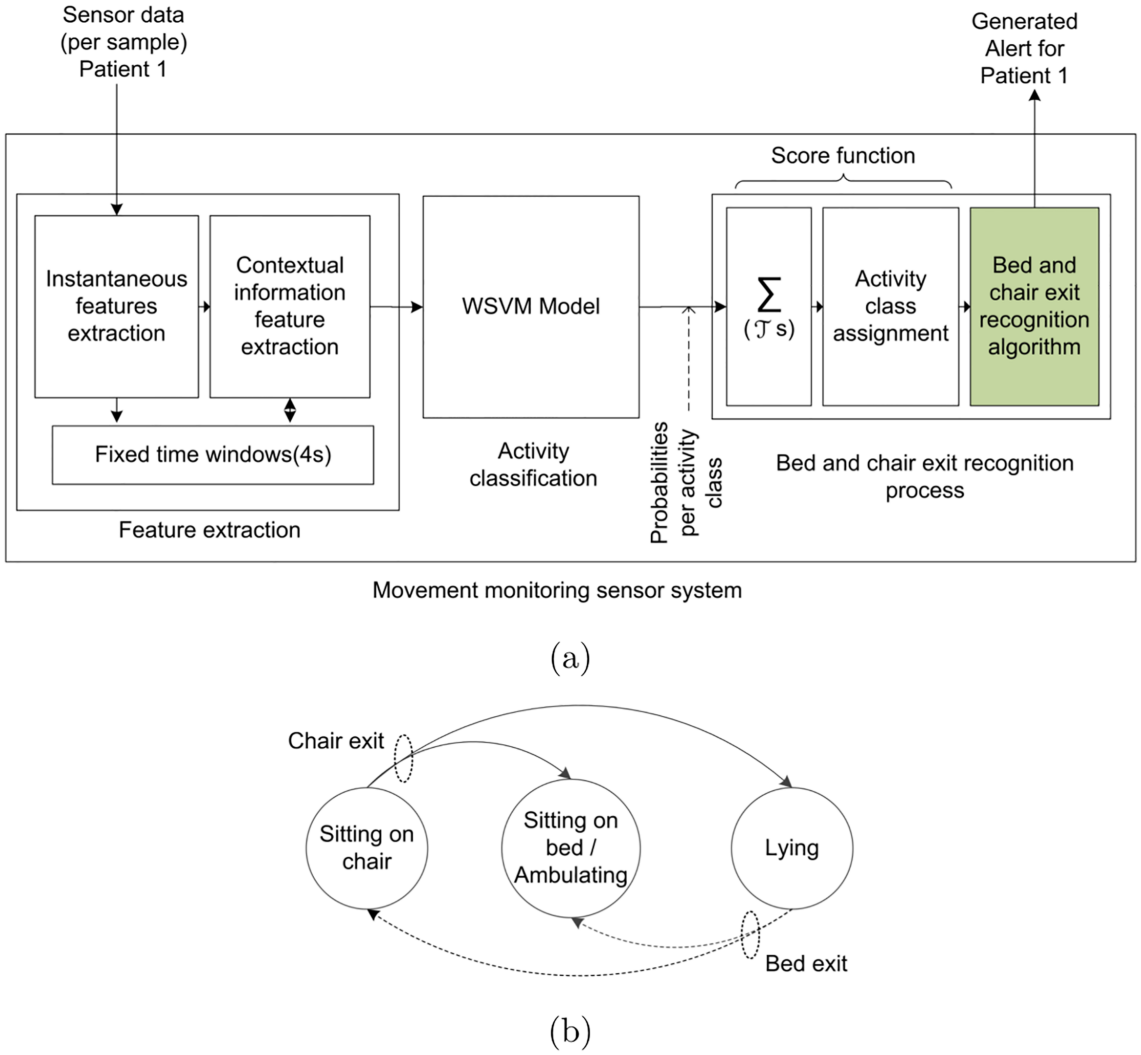
Proposed movement monitoring sensor system. (a): bed and chair exit recognition system and (b): patient state transition model used by the bed and char exit recognition algorithm (green block in (a)).

Although the use of a passive wearable sensor approach provides the distinct advantages of lightweight, low cost, and battery-free; a limitation originates in the wireless powering, as it depends on the sensor gathering sufficient power from EM signals from RFID reader antennas for powering W^2^ISP circuitry and backscattering data from the sensor. Hence, the effects of variable distance to RFID reader antennas, destructive interference due to multipath, radio frequency band interference and occlusion by RF opaque objects such as the human body cause irregular, incomplete and noisy readings that are delivered to the movement monitoring sensor system (see Fig 4).

#### Movement monitoring sensor system

The proposed system considered activities of high risk of falling that are performed by older patients without supervision in a hospital room context [31]. Previous research [31] has determined that exiting the bed and exiting the chair are high falls-risk movements that have to be identified for falls preventing falls. Hence, we proposed a movement monitoring sensor system, shown in Fig 4(a), that consists of three main stages: i) feature extraction; ii) activity classification; and iii) bed and chair exit recognition process.

Feature extraction refers to obtaining essential information from the sensor and RFID data stream from which the activity classification stage, based on the class-sensitive classification method of weighted support vector machine (WSVM), can accurately classify the performed activity. The present work has considered a set of activities (classes) representative of those performed by patients in a hospital ward room setting to support our wearable falls prevention intervention. As a consequence, we considered the activities: i) Sitting-on-bed; ii) Sitting-on-chair; iii) Lying; and iv) Ambulating. Although the number of activities are naturally limited by the context of our application, there is clear intra-class diversity, for example, patients lying on bed were watching TV, resting or reading; similarly, during ambulation a patient can be either walking with or without a walking aid. The bed and chair exit recognition process collects the output of the WSVM model, assigns an activity to each sensor observation using a score function and generates an alert using the bed and chair exit recognition algorithm in the event a bed or chair exit is recognized following the patient state transitions described in Fig 4(b).

### Data collection

#### Study participants

We trialled a cohort of hospitalized older people, inpatients in the geriatrics ward of the Queen Elizabeth Hospital in Adelaide, South Australia. The patients were 71 years and older, a male to female ratio of about 1 : 2, with no cognitive impairment and with the ability to mobilize independently or use a walking aid. The patients were recruited directly from the hospital, informed written consent was obtained and no honorarium was provided. We trialled 26 patients in our pilot study. However, data related to three patients were discarded from the performance evaluation study because of poor signal collection from the sensor caused by a false contact at an antenna junction point due to mechanical wear and tear. The mechanical failure is because of the laboratory prototype nature of the sensor developed for the study. Therefore, data from 23 patients were used to evaluate the performance of our system; patient’s data is available at S1 Dataset. However, the 26 patients participated in two surveys which included one incomplete post trial survey.

The patients were informed of the activities contained in the script to ensure that they had no objections to any of the proposed activities but were not informed of the order before the trial start.

#### Hospital settings

The study was undertaken in the rooms of each patient; rooms were single or double bed, hence the room dimensions in Fig 2(b) are approximate. Although every room was different, beds and chairs and their respective setting were uniform for all trials. Our investigation in [35] confirmed that in a clinical setting it was possible to achieve high performance for activity classification using three antennas focused on areas of interest rather than more antennas covering a wider area. Therefore, this study in a hospital setting used a deployment with two antennas located on ceiling level inclined towards the bed and one antenna on the wall opposite the chair inclined towards it as shown in Fig 2(b); the bedside chairs were located at either left or right side of the bed and the patient always exited the bed on the side of the bed that was next to the chair. No other furniture was added or removed, and overbed tables were placed outside the walking route during the trials.

The patients performed a set of scripted lists of activities of daily living (ADL) that included walking to the chair, sitting on the chair, getting off the chair, walking to the bed, lying on the bed, getting off the bed and walking to the door. The patients were instructed at the beginning of the trial to perform each activity at their own pace and as comfortable as possible; no other instruction was given as to how to perform each activity. The patients were also instructed to request a trial termination if they were distressed or in discomfort. During the trials two members of the research team were present, the first member instructed the patients with the activities that needed to be performed from the script and annotated the activities being undertaken, the ground truth, in the sensor data capturing software; the ground truth was later contrasted with the activities determined by our proposed method to measure the system’s performance. The second member was present to ensure the safety of the patient due to their frail condition and could interrupt at any time.

#### Hospital trial procedure

Each patient was trialled in his/her respective room. Patients only performed the ADLs described above, which were repeated two or three times, depending on the patient’s ability to continue the trial. The duration of the trials for this cohort was about 20 to 25 minutes per patient and was performed during the day between 2 pm and 4 pm.

The patients were required to complete a pre-trial survey and two post-trial surveys, immediately before and after the trial respectively, to measure the changes of perception and their level of acceptance of the system.

During trials, patients were not always lying flat on the bed. Patients in their bed had the position of the movable head rest of the bed either flat or inclined as patients were resting, receiving visits, watching television or reading. Hence, during the trials, multiple postures in bed were captured when the patient was lying on the bed. Moreover, all the patients that participated in the trials when sitting on the bed, sat with legs off the bed as opposed to sitting straight up with their legs still on the bed; thus we consider a patient sitting with legs off the bed as having the intention of getting out of bed after a period of lying on the bed.

#### Acceptability study survey design

Two surveys were designed to evaluate equipment acceptability based on the Sensor Acceptance Model developed by Fensli et al. [43]. The first survey is based on the “pre-trial expectation” factor identified and used by Fensli et al. [43]. The questions measured the perception of the patients about the system to recognize bed and chair exits and their apprehension towards the use of the equipment. Unlike in Fensli et al. [43] where the first survey was only administered before the trial, in our study the survey was completed by each patient before and after their trials to enable the measurement of change in perception.

The second survey was completed after each trial to measure acceptability and privacy concerns perceived by the users. The Sensor Acceptance Model [43] identified five key factors (hygienic aspects, physical activity, skin reactions, anxiety, equipment) to determine the patients’ acceptance of wearable sensor based systems worn in contact with the skin. We selected three of the factors identified by Fensli et al. [43]: “physical activity”, “anxiety” and “equipment” as they are relevant to our study and our sensor is not worn against the skin. We added “privacy” as an acceptability factor due to the importance of privacy violation concerns identified in previous studies [19]. The questions were formulated as positive or negative statements and used an eleven point semantic differential scale (0–10) where answers indicated agreement/disagreement and no-problem/problem. Both surveys are shown in the Results section, where a score of 10 indicates full agreement or satisfaction with the system.

### Data processing

#### Feature extraction

**Fig 5:**
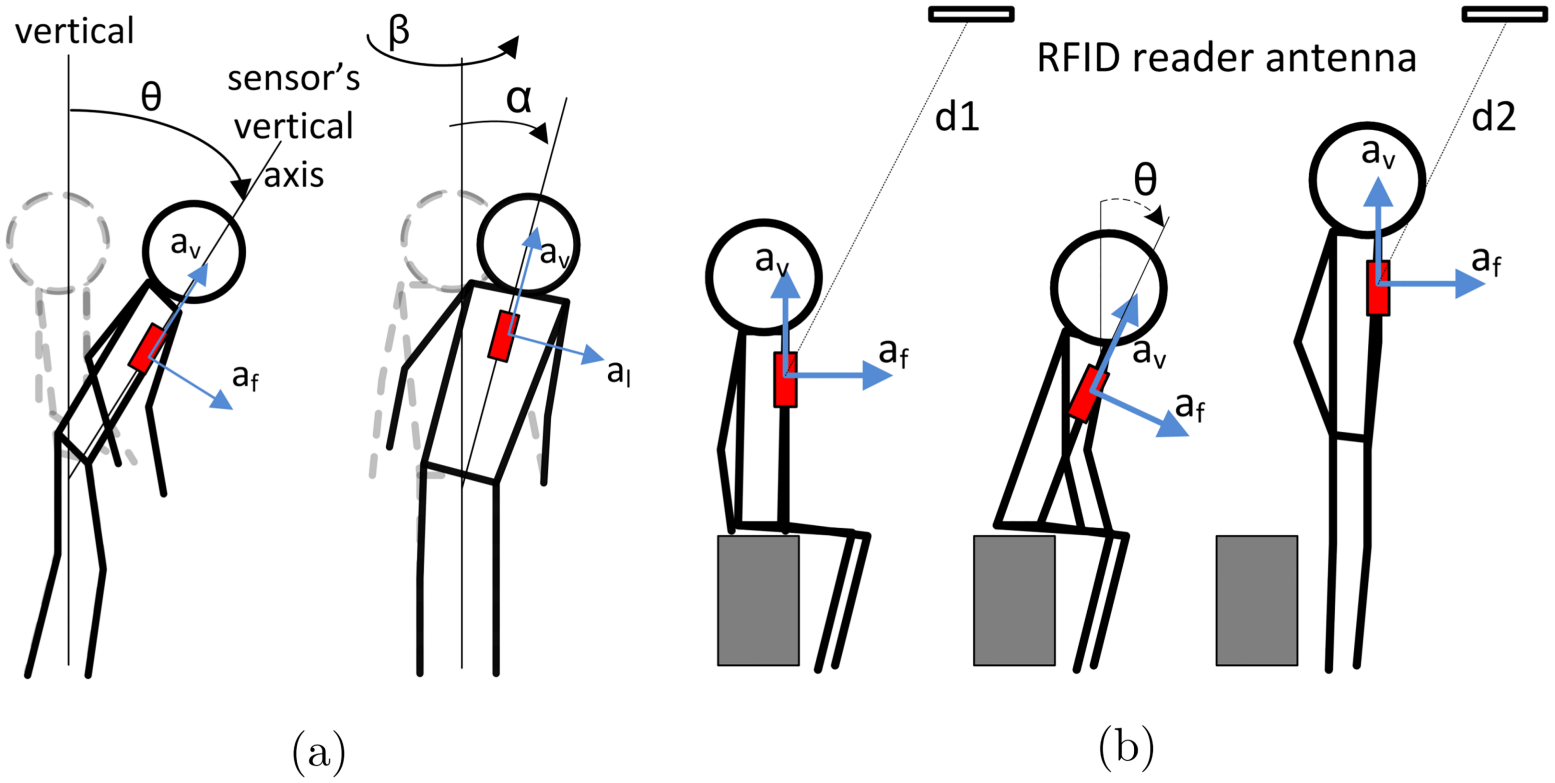
Body motion of person wearing W^2^ISP. (a) body rotational angles for a person using the W^2^ISP (in red) that are approximated using acceleration information; (b) sequence of sitting to standing showing distance to antenna, where distance to a person standing (d2) is shorter to when the person is sitting (d1); this also means that RSSI readings are higher when the person is closer to the antenna (standing).

This stage generates from the sensorial data the features engineered to capture the underlying body motions as input to the activity classification stage illustrated in Fig 4. Each reading, *v*_*i*_ = (*a*_*f*_, *a*_*v*_, *a*_*l*_, *RSSI*, *aID*, *F*, *ϕ*) where i∈N we obtain, contains information from the sensor and RFID infrastructure described previously in Section Wearable Sensor Technology. We are interested in RSSI as its measurements are proportional to 1/*d*^4^ where *d* is the line of sight distance between a sensor and an RFID antenna [44]. The hypersensitivity of RSSI to distance implies that patterns in changes in RSSI values with relation to a given RFID antenna can potentially help discriminate between postures, such as sitting and standing as well as a change in posture from sitting to standing—see Fig 3(b), that may not be noticeable using acceleration information alone. This is because as acceleration measurements are often similar for sitting and standing postures where the body trunk is likely to be upright as shown in Fig 5(b). We also use RF phase (*ϕ*) as previous studies [42, 44] have demonstrated that phase measurements computed by an RFID reader based on a received signal from an RFID transponder provides spatial information such as a tag’s distance from an RFID antenna, and velocity. Therefore we include RF *ϕ* based features that can potentially capture movements and activities performed by hospitalized patients wearing the sensor. In addition to sensor information *v*_*i*_, we also use the patient’s gender and the timestamp *t*_*i*_ at which the sensor reading was captured. Hence we consider a sensor reading as the *x*_*i*_ = (*t*_*i*_, *gender*, *v*_*i*_) from which we derive two types of features: i) instantaneous features; and ii) contextual information features.

#### Instantaneous features

These features are obtained directly from the most recent sensor reading and provide useful information related to the patient and the action being performed as captured by the accelerometer sensor and the RFID infrastructure. For every sensor reading, now defined by *x*_*i*_ = (*t*_*i*_; *gender*; *v*_*i*_), we calculate a set of features directly derived from the values observed in *v*_*i*_, i.e. instantaneous features, described in Table 1. In addition to considering elements of *v* as features (i.e. *a*_*f*_, *a*_*v*_, *a*_*l*_, *RSSI*, *aID*); we also consider the resultant acceleration (feature item 6), the sine of body tilting angle (*θ*) [26], and body rotation angles yaw (*β*) and roll (*α*) determined as shown in Fig 5(a). Given that the W^2^ISP only includes an accelerometer, these angular measurement values were approximated from the raw accelerometer readings (feature items 4, 9 and 10 in Table 1). We also consider the time difference with the previous reading Δ*t*_*i*_ = *t*_*i*_ − *t*_*i*−1_ as the rate of received sensor readings are irregular i.e. Δ*t*_*i*_ ≠ Δ*t*_*i*−1_ and can provide potential information based on the EM illumination levels of the sensor based on its orientation and distance from the studied fixed antenna configuration.

**Table 1:** List of instantaneous features extracted of interest to system. (* indicates feature used.)

Features		Description
1.	Frontal acceleration (*a*_*f*_)*	Dorsoventral axis acceleration values in *g* (i.e. range −1:1)
2.	Vertical acceleration (*a*_*v*_)*	Anteroposterior axis acceleration values in *g*
3.	Lateral acceleration (*a*_*l*_)	Left-right axis acceleration values in *g*
4.	Sine of body tilting angle (pitch)*	Sine of body tilting angle (*θ*) towards the front or back with respect to vertical in the midsagittal plane [26], approximated as: $\sin(\arctan\left(\frac{a_{f}}{a_{v}}\right))$
5.	Received signal power (RSSI)*	Received signal power from the sensor as received at reading antenna. RSSI is calculated as: $P_{t}G_{t}^{2}G_{path}^{2}K$ where *P*_*t*_ is the output power of the RFID reader, *G*_*t*_ is the RFID antenna gain, *K* is the W^2^ISP backscatter gain and *G*_*path*_ is the one-way path gain of the deterministic multipath channel [44] which is inversely proportional to the square of the direct path distance (i.e. $d_{0}^{2}$). Hence, RSSI is inversely proportional to $d_{0}^{4}$.
6.	Resultant acceleration*	Magnitude of acceleration vector given by $a_{t}=\sqrt{a_{f}^{2}+a_{v}^{2}+a_{l}^{2}}$.
7.	Time difference Δ*t*	Time difference with previous sensor observation (regardless of receiving antenna).
8.	Participant’s gender*	
9.	Trunk yaw angle*	Rotational angle from dorsoventral axis, approximated as: $\beta \approx \arctan(\frac{a_{l}}{a_{f}})$
10.	Trunk roll angle*	Tilting angle in the coronal plane, approximated as: $\alpha \approx \arctan(\frac{a_{l}}{a_{v}})$
11.	Antenna ID (*aID*)	RFID antenna receiving current tag reading.

#### Contextual information features

These features are generated from segments obtained from partitioning the time series of sensor readings. We generated contextual information features since a single sensor reading is insufficient to capture adequate information regarding underlying activities of duration longer than a single sensor reading and to benefit from information in the temporal vicinity of an activity. Hence, contextual information features overcome the limited ability of instantaneous features to capture information. In general, these features provide an insight into the physical motions and changes in location of a patient by using information from the recent past sensor readings in a segment. In previous research [45] we found that using a partitioning or segmentation approach for extracting contextual features using a fixed time window method improved the performance to be better or similar to more complex segmentation methods; moreover fixed time window based segmentation method is easily implemented. Here, limiting the size of the segment is important as events distant in time become less relevant to the current activity.

More formally, we define a segment as $S_{\left[t_{i}-T,t_{i}\right]}=\left\{\left(t_{o},gender,v_{o}\right)\right\}{|}_{o=t_{i}-T}^{t_{i}}$ which contains all received sensor readings during a period of *T* seconds from the sensor observation at time *t*_*i*_ where *t*_*i*_ − *T* < *t*_*i*_. Using these sensor readings within the segment *S*_[*t*_*i*_−*T*, *t*_*i*_]_, we calculate the contextual features which are shown in Table 2. Subsequently, these features are calculated for the next received reading at *t*_*i*+1_ and for every *t*_*j*_, where *j* > *i*. We have used *T* = 4s in our work.

**Table 2:** List of contextual information features extracted of interest to system. (* indicates feature used, p.a. = per antenna.)

Features		Description
12.	Readings p.a.*	Indicator of number of readings per antenna in the segment, in our case we consider antenna2.
13.	Antenna collecting maximum power	ID of antenna (aID) with maximum received power in segment.
14.	Antenna collecting minimum power*	ID of antenna (aID) with minimum received power in segment.
15.	Vertical displacement* (*d*)	Cumulative body displacement in the sensor’s vertical axis during a segment, calculated as: $d=\overset{\delta t}{\underset{0}{\;\int \int \;}}a_{v}dt^{2}$.
16.	Mutual information of bed and chair areas* (*m*_*bed*−*chair*_)	Events occurring between these two areas given by the number of consecutive readings captured in both bed and chair areas, calculated as: $m_{bed-chair}=\frac{1}{n}\sum_{i=1}^{n-1}\left(1_{\left[\left\{bed,chair\right\}=\left\{ant_{i},ant_{i+1}\right\}\right]}+1_{\left[\left\{chair,bed\right\}=\left\{ant_{i},ant_{i+1}\right\}\right]}\right)$; where $1_{x}$ assumes 1 if *x* is true and 0 otherwise and *ant*_*i*_ refers to the antenna receiving the *i^th^* sensor reading in a 4 s segment with *n* sensor readings, used in [45]. In our case we use bed and chair antenna pairs (antenna3, antenna1) and (antenna2, antenna1).
17.	Pearson correlation coefficient* (*r*)	Correlation between axes information, calculated as: $r_{a,b}=\frac{1}{n-1}\sum_{i=1}^{n}\left(\frac{a_{i}-\bar{a}}{s_{a}}\right)\left(\frac{b_{i}-\bar{b}}{s_{b}}\right)$; where we considered *a*, *b* = {*a*_*v*_, *a*_*f*_}, *a* ≠ *b* and *s*_*x*_ is the standard deviation of the samples *x* in the window.
18.	Mean and standard deviation of RSSI*	Mean and standard deviation of received power received per antenna during 4 s time window. In our case, we consider antenna1 and antenna3.
19.	Median, sum of absolute value and standard deviation of CFPR*	Constant Frequency Phase Rate (CFPR) defined as *CFPR* = *ϕ*_*aID*, *F*_(*i*) − *ϕ*_*aID*, *F*_(*i* − 1), for each antenna. Measured during a 4 s segment as defined in [42]. We consider median of antenna1 and antenna3; absolute value of antenna2 and standard deviation of antenna2 and antenna3.
20.	Standard deviation of VFPR*	Variable Frequency Phase Rate (VFPR) defined as $VFPR=\frac{\phi (i)-\phi (i-1)}{F(i)-F(i-1)}$, for antenna3 during a 4 s segment; features as defined in [42].
21.	Mean and standard deviation of acceleration*	Mean and standard deviation of acceleration values during 4 s time window; we consider the mean of *a*_*v*_, *a*_*f*_, *a*_*l*_ and standard deviation of *a*_*l*_.

In particular, we considered readings captured by each antenna (feature item 12) and mutual information from bed and chair areas (feature item 16) as developed in [45]. We also calculate features that can provide information about the variations of movement by the patients as provided by the values in *v*_*i*_; for example, we consider the Pearson correlation coefficient (*r*), the approximate displacements from the acceleration component of the sensor’s vertical axis *a*_*v*_ and the mean and standard deviation of acceleration values within the segment (feature items 15, 17 and 21). We also consider features that provide insight into the variations in RSSI; we consider the mean and standard deviation of RSSI as well as identify the antennas that capture minimum and maximum RSSI values within the segment (feature items 13, 14 and 18). Furthermore, we consider the features engineered in [42] which use phase information to determine possible small scale movements of the patient (feature items 19 and 20).

We performed feature selection to eliminate those features that are less relevant to our activity classification problem without loss of information. From the complete feature vector that considers instantaneous and contextual information features, we performed feature selection using the WEKA data mining tool [46]. We selected two simple statistical classifiers—logistic regression and Bayes network—and eliminated features that were low ranked for both methods. The features selected are indicated in Tables 1 and 2.

#### Activity classification

The activity classification stage is based on the machine learning method of weighted support vector machines (WSVM) [47], a classifier based on SVM [48] originally designed for a two-class problem (binary). In SVM, the model treats all training samples with equal importance, and this can lead to misclassification in the case of imbalanced data as is our case. Class imbalance is inherent to our problem because some activities performed by people are of longer duration than others; for example, a hospital patient will spend more time lying in bed than ambulating. Moreover, data is more easily collected from some activities than others; for example activities closer to the RFID antenna are easier to collect than those performed farther from the RFID antennas, also the sensor can be occluded from RFID antennas by the patient’s body during some movements affecting the sensor’s powering and data collection.

Given the training data $D_{l}={\left\{\left(x_{i},y_{i},s_{i}\right)\right\}}_{i=1}^{l}$, where each training sample *x*_*i*_ is a feature vector associated to a two-class label *y*_*i*_ ∈ {−1, +1}, WSVM, as opposed to SVM, treats each observation *x*_*i*_ differently according to its known weight $s_{i}\in R$. The classification model *w*, is learned by minimizing the convex objective function:

$$\begin{array}{cc} \text{minimize} & \Phi (w)=\frac{1}{2}{\left\|w\right\|}^{2}+C\sum_{i=1}^{l}s_{i}\xi_{i}\\ \text{subject}\;\text{to} & y_{i}\left(\langle w,x_{i}\rangle +b\right)\geq 1-\xi_{i},i=1,\cdots ,l\\ & \xi_{i}\geq 0,i=1,\cdots ,l \end{array}$$ (1)

where *C* is a constant determined by model parameter selection (see Section Statistical Analysis), *ξ*_*i*_ are error margins and *b* is the offset of the hyperplane from the origin. Weights *s*_*i*_ are only considered during the training stage as they are used to affect the model *w* treatment of each individual class; the values of *s*_*i*_ are not determined autonomously by WSVM during training, but are parameters to the model.

Nonetheless, there are two main limitations in using this model in our problem. The first limitation is that our problem requires a multiclass classification model given our set of activities i.e. $y_{i}\in K=\left\{\text{Sitting}-\text{on}-\text{bed},\;\text{Sitting}-\text{on}-\text{chair},\;\text{Lying,}\;\text{Ambulating}\right\}$. We use a one-against-one method for multiclass SVM as it has been demonstrated to perform better than other methods for multiclass SVM [49]. In our study, we implemented the multiclass WSVM model from LIBSVM [50].

The second limitation is that weights are unknown at the time of training. Therefore, we formulate a second optimization problem to find the best set of weights $s_{i}^{k}$ for k∈K that maximizes the overall F-score for the training set. We use covariance matrix adaptation evolution strategy (CMA-ES) [51], a state-of-the-art evolutionary algorithm, for optimization of the set of weights. This method determines the optimal solution by iteratively estimating an optimal set of parameters from an initial set of weights. In our study, we used the software of [51] to optimize the set of class-wise weights. We chose the best set of weights $s_{i}^{k}$, during model parameter selection (see Section Statistical Analysis), after analyzing 200 random sets of initial weights.

#### Bed and chair exit recognition process

In this stage, an alert signal is triggered on the occurrence of a recognized bed or chair exit. These exiting events from both bed and chair occur in a common area around the bed and the chair as illustrated in Fig 2(b). Hence, the intervening staff is directed to the same area of the bed and chair.

To evaluate the occurrence of bed and chair exits, we propose a score function that first sums the estimated classification probabilities for each activity (class), considered as normalized scores per activity, over a non-overlapping time window of duration T of data produced from the activity classification stage. The goal of the score function is to assign an activity class that is dominant in the time window T and is therefore less affected by activity classification errors from the underlying WSVM model. The score function then selects the activity class with the highest sum and assigns that activity to the complete data window T.

The scoring function mitigates the resulting effect of those misclassified sensor readings that could produce undesirable false alarms if they were considered in the decision to issue an alarm without the score function. The score function reduces possible false alarms by evaluating the dominant class label of multiple sensor readings over a short time period as a more accurate representation of the activity being performed to generate an alarm signal. We have considered a fixed time window approach of duration T. We consider the value of T in the range of 0.1 to 5.0 s where the limit value of 5.0 s is more than double the minimum time a posture transition takes place [34] so that a complete transfer is included in a single window. The value of T was chosen during model parameter selection (see Statistical Analysis) such that it maximizes the overall F-score.

When a bed or chair exit has been identified by the algorithm, an alert signal is issued as detailed in Fig 4(b). Exits from a bed are detected when the classified activity of Lying-on-bed is followed by a Sitting-on-bed or Ambulation. In the case of exits from a chair, we identify such an event when a Sitting-on-chair activity is followed by any other activity. After an alert is issued, we consider that it is physically impossible for another alert to occur in the next 1.75 s, which corresponds to the minimum time for a posture transition to take place and longer periods can possibly overlap with other valid posture transitions. Hence, we cancel any additional alerts within that period. Pseudocode is presented in Supporting Information S1 Pseudocode.

#### Statistical analysis

**Fig 6:**
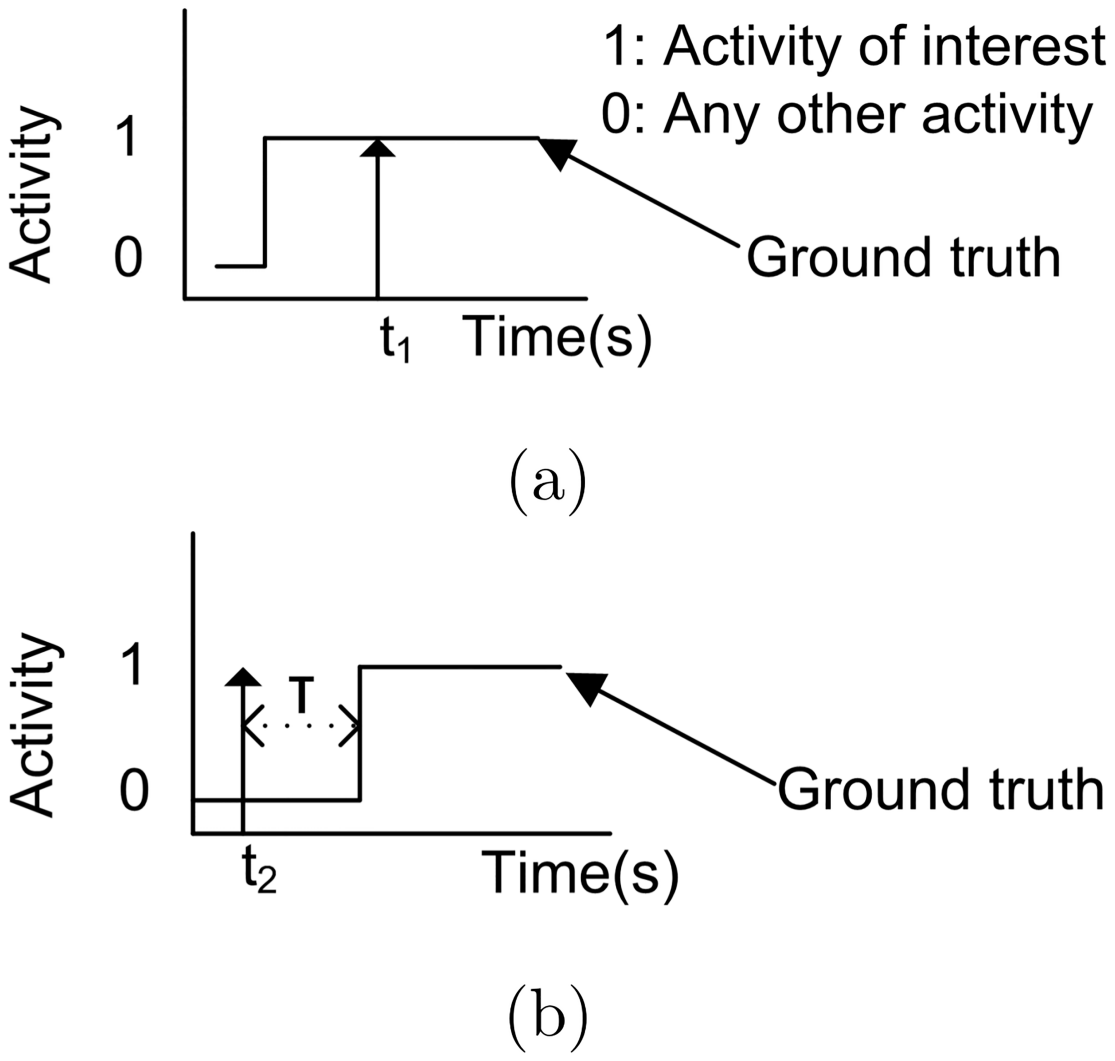
True positive of bed and chair exit recognition. A TP of bed and chair exit is said to occur when an alert (arrow) is triggered at t_1_ and t_2_ for: (a) a real activity is occurring; (b) real activity occurs less than 5 s after an alert.

In this study, true positives (TP) were correctly recognized bed and chair exit alerts when: i) the alert occurs when the person is actually performing an activity of interest as illustrated in Fig 6(a); or ii) the real activity (ground truth) occurs no more than a time *T* = 5 s after the alert signal as illustrated in Fig 6(b). False positives (FP) or false alarms are recognized bed and chair exits that do not follow the TP criteria, i.e. incorrectly identified as target activity. False negatives (FN) are unrecognized bed and chair exits (misses).

We evaluate the performance of the proposed system using recall (also known as sensitivity in the literature), precision and F-score, the harmonic mean of recall and precision, as these metrics consider the occurrence of errors (misses and false alarms) in relation to true alarm events (TP). These are defined as: i) recall = TP/(TP+FN); ii) precision = TP/(TP+FP) and iii) F-score = (2×precision×recall)/(precision+recall).

We consider evaluating our approach using a leave-one-out cross validation because this approach gives a clear indication of the performance of the system when tested with data from a patient not known to the algorithm, as is the case in a practical deployment of the system in a hospital. In this validation process, the data of a patient is used for testing (testing set); the data of a different patient is used for parameter validation (validation set) and the rest of the patients’ data (21 patients) is used for training the system (training set). This process is repeated sequentially where each of the 23 patients are selected one by one for testing. Hence, the data of the patient used for testing is always unknown to the system as it was trained with data of other patients. We performed model parameter selection using the validation set, where the model with the highest F-score was selected for testing.

We compare performance metrics (precision, recall, F-score) of the movement monitoring sensor system with other methods using an independent *t*-test where our null hypothesis (*H*_0_) is that the performance of our movement monitoring sensor system and other tested method are the same. To compare the pre- and post-survey responses, we use the Mann-Whitney *U*-test, a non-parametric test adequate for evaluating ordinal non-normal data as is the case of our surveys [52]. The non-normality of the data was confirmed by the using the Shapiro-Wilk test. The null hypothesis (*H*_0_) of the Mann-Whitney *U*-test is that there is no difference between the tested sets of data.

## Results

### Bed and chair exit recognition system performance results

We used sensor and RFID data from 23 patients as three patients were removed as explained in Section Data Collection—Study Participants. Recognition of bed and chair exits, shown in Table 3, illustrates the results for our windowing method for all trialled patients. We used a fixed time window of duration T = 4.8 s where the overall F-score metric is > 72%, as shown in Table 4. We also compare with our study in [36], where we used a dynamically weighted CRF model [53] to classify activities for the recognition of bed and chair exits as in Fig 4(b), using the features developed in this research to make a fair comparison. We have determined that the median delay for the recognition of bed and chair exits is of 4.09 s; also the mode of all delays, rounded to the nearest second, is of 4 s. This indicates that the majority of recognized alarms are not generally longer than the duration of the time window used for determining a bed and chair exit alarm.

**Table 3:** Recognition of bed and chair exits in the hospital setting. TP = true positives, FP = false positives or false alarms and FN = false negatives or misses.

Patient ID	Total bed & chair exits	Fixed time window T=4.8s			Method of [36]		
		TP	FP	FN	TP	FP	FN
p.1	5	2	3	3	2	28	3
p.2	6	4	3	2	5	5	1
p.3	5	4	2	1	5	1	0
p.4	4	4	0	0	4	13	0
p.5	6	6	4	0	5	3	1
p.6	4	3	3	1	3	4	1
p.7	2	2	0	0	2	3	0
p.8	3	3	1	0	3	3	0
p.9	6	5	2	1	3	3	3
p.10	6	3	5	3	2	12	4
p.11	3	2	0	1	2	0	1
p.12	5	3	6	2	5	7	0
p.13	6	6	6	0	6	46	0
p.14	5	4	2	1	4	4	1
p.15	6	6	1	0	6	22	0
p.16	7	5	3	2	6	4	1
p.17	3	2	2	1	2	1	1
p.18	4	4	0	0	4	4	0
p.19	6	4	3	2	5	16	1
p.20	6	4	3	2	6	33	0
p.21	6	6	3	0	6	9	0
p.22	6	6	3	0	6	4	0
p.23	5	5	1	0	5	19	0

**Table 4:** Performance metrics for tested methods. Including fixed size window and the method of [36] modified for bed and chair exit detection. Results in %.

	Recall	Precision	F-score
Fixed time window	81.44 ± 18.89	66.82 ± 20.24	72.48 ± 17.84
Method of [36]	84.67 ± 20.69	42.80 ± 23.60	52.75 ± 21.09

### Acceptability study results

**Fig 7:**
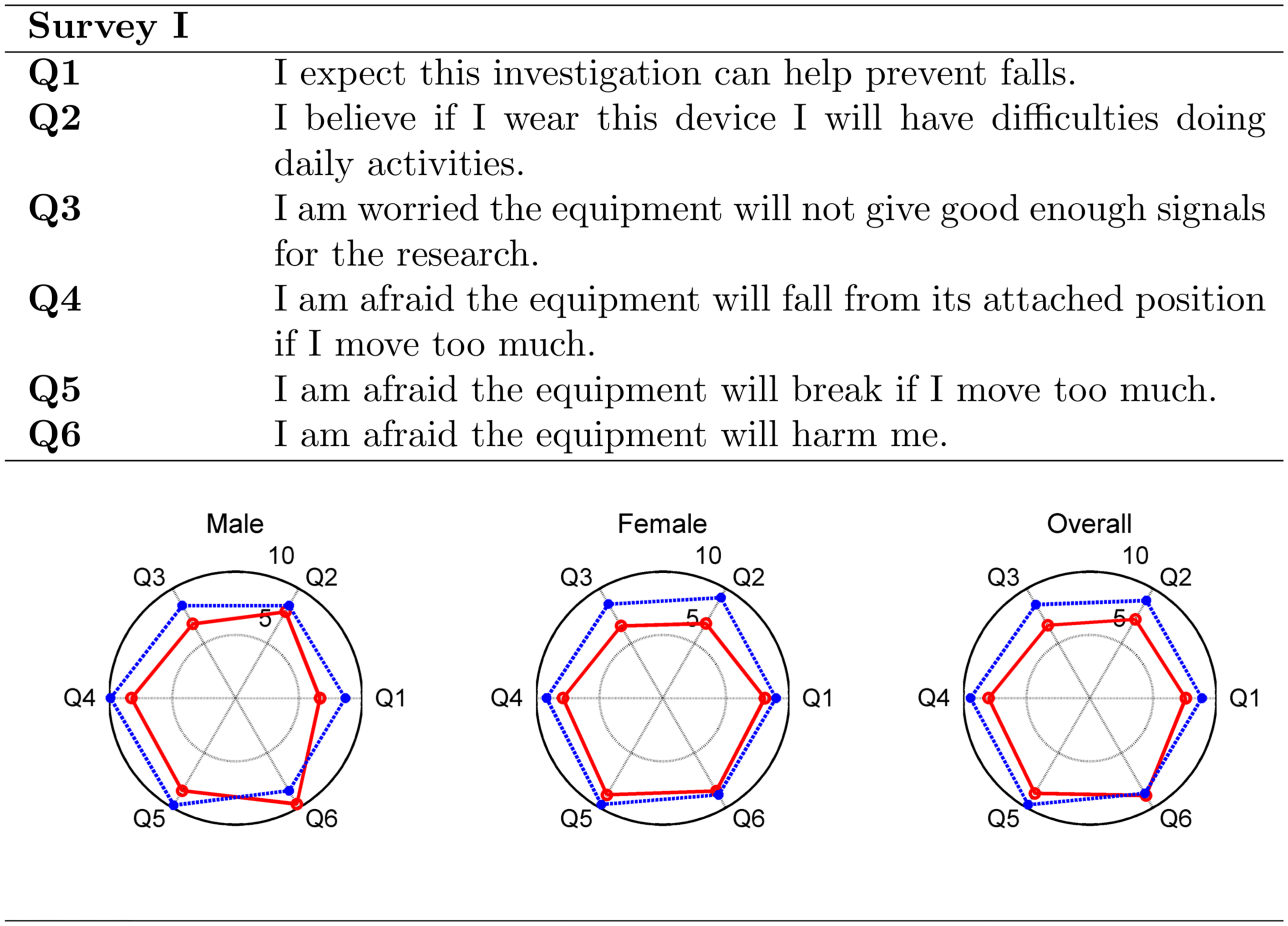
Results from first survey. Radar charts of pre-trial (red line) and post-trial (blue line) responses to the first survey, showing average scores for overall and male and female participant cohorts. Score range from 0 to 10, where 10 indicates full satisfaction with the equipment.

Twenty six patients participated in the surveys, with one patient that partially completed the second post-trial survey. Analysis of the first survey responses in Fig 7, where each radial axis indicates the mean score of a question, indicates in general an improved perception of the sensor equipment by the patients after participating in the trial. The pre-trial assessment of the system had an overall average score of 7.83 with partial overall scores ≥ 6.65 as shown in Fig 7 (red line). On the other hand, the post-trial assessment achieved an overall average score of 9.03 (larger outer plot with scores higher than the smaller inner plot). However, patient’s pre and post-trial response score to question Q6: “I am afraid the equipment will harm me” had a marginal overall score decrease of 0.2.

Comparing pre and post-trial response scores using Mann-Whitney test (see Section Statistical analysis), for overall results, show that the medians for questions Q2 (*U* = 225.5, *P* = 0.019), Q3 (*U* = 215.5, *P* = 0.016) and Q4 (*U* = 237.5, *P* = 0.022) are statistically significantly different. Considering gender differences in our patient group, the difference in response among male participants were not statistically significant (*P* > 0.08). However, the female participants’ pre and post-trial response differences for question Q2: “I believe if I wear this device I will have difficulties doing daily activities” (*U* = 90.5, *P* = 0.030), and Q3: “I am worried the equipment will not give good enough signals for the research” (*U* = 84, *P* = 0.023) were statistically significant. For all other questions, the median differences were not significant (*P* ≥ 0.11) although having consistently higher average scores in the post trial responses.

**Fig 8:**
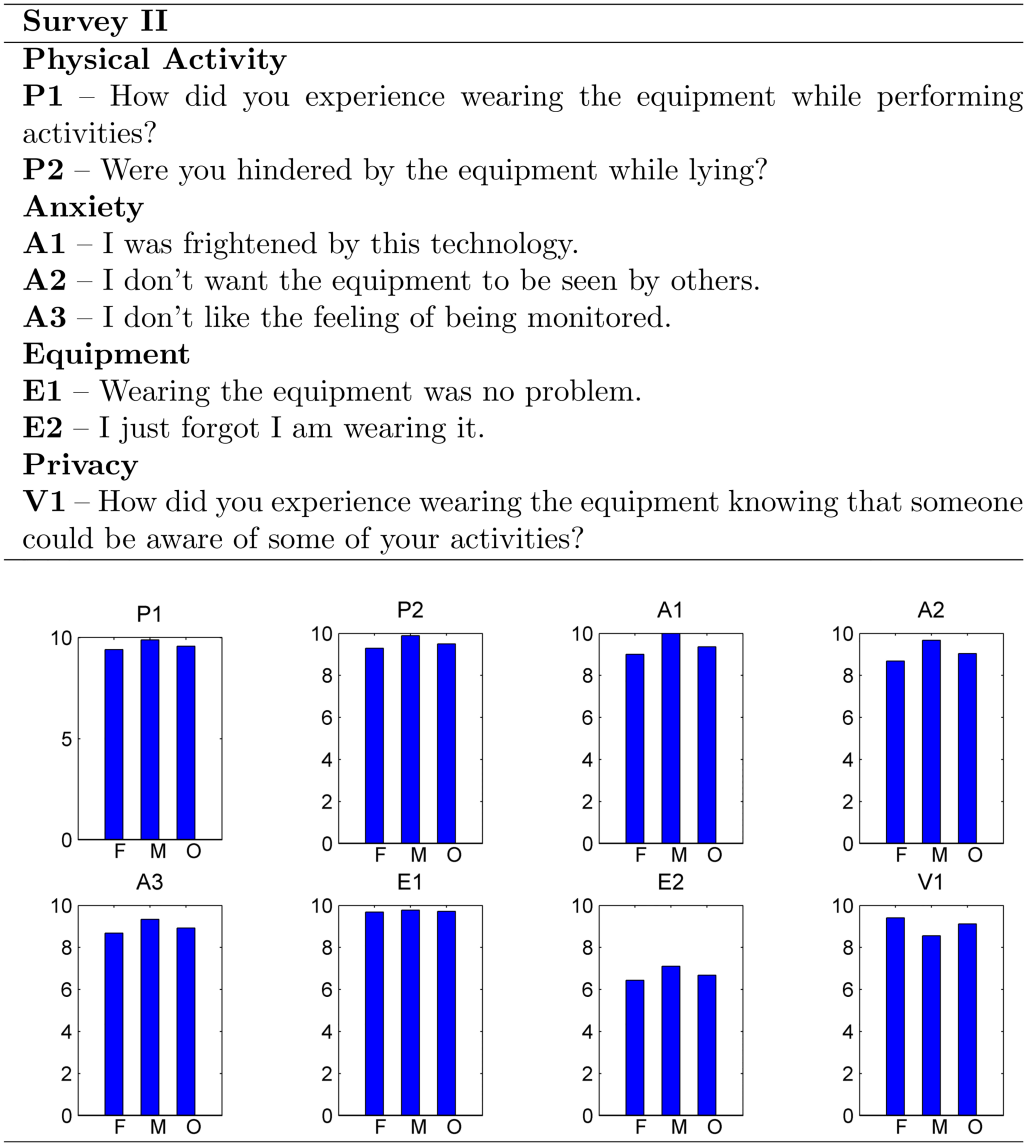
Results from second survey. Second survey conducted post-trial only, showing average score responses in the bar graph. F: female, M: male O: overall.

Analysis of the second survey shown in Fig 8 indicates an overall average score of 8.99 for all four factors (≥ 6.68 overall) of the Sensor Acceptance Model (physical activity, anxiety, equipment and privacy). The lowest score was for question E2: “I just forgot I am wearing it” in the overall, and gender based analysis, demonstrating an overall concern for the high visibility of the sensor during the trials.

## Discussion

This pilot study to evaluate a new technological intervention for preventing falls in hospitals and nursing homes completed with frail hospitalized older people suggests that it is possible to undertake the monitoring of movements associated with bed and chair exits using a single batteryless sensor loosely worn over clothing by a patient. Furthermore, the sensor was found to be acceptable to the hospitalized patients that participated in this pilot study.

### Movement monitoring sensor system

This research builds on previous studies from our group focused on bed exits [34–36] in healthy old and young people as opposed to hospitalized older people. In the current study, we adopted a 3 antenna deployment since this arrangement performed well in [35, 36] for bed exit detection. It is important to undertake research within hospitalized older people who are frail as, unlike healthier cohorts, hospitalized older people perform shorter ambulations and move differently when exiting beds and chairs. For example, during our trials, they made several attempts before successfully exiting a bed and their postural changes occurred much more slowly. In addition, furniture tend to be placed in close proximity of each other given the illness and frailty of hospitalized patients and their limited mobility limiting the time patients ambulate between, for example, the bed and the chair. We therefore applied classification methods that consider the effects of minority classes, present in our data because of short-duration activities and reduced sensor data collection during postures where the sensor is occluded. We used a score function based on the use of a fixed time window to determine classes and generate alarms to reduce errors from possible misclassifications.

**Fig 9:**
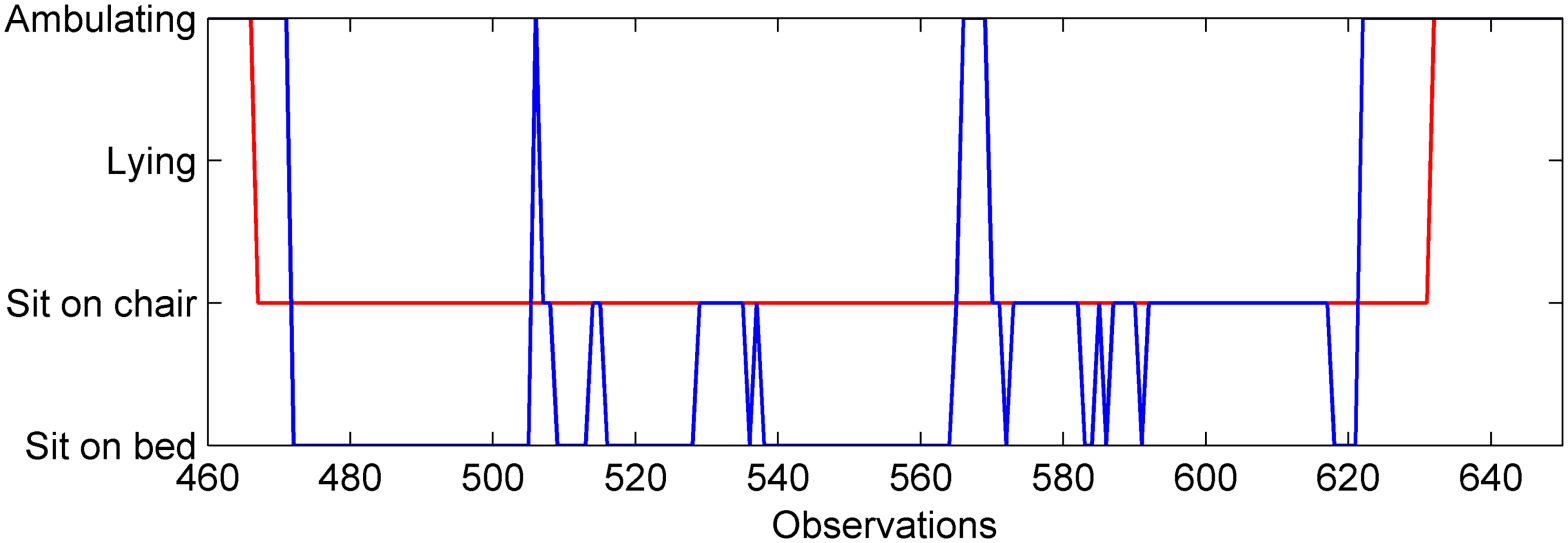
Classified activity contrasted with the ground truth for a patient near a chair. In red: ground truth, in blue: classified activity.

Although our results demonstrate, for the first time to the best of our knowledge, the possibility of using a batteryless wearable sensor to detect bed and chair exits in hospitalized older people, the resulting precision is below expected and thus the number of false alarms (FPs) remains relatively high. This is shown in Table 3 where some of the FPs are caused by the classifier not being able to discriminate among sitting-on-chair, sitting-on-bed and ambulating in the space between a bed and a chair. This is caused by lack of sensor observations during transitions, which are usually of short duration as the chair is placed next to the bed as shown in Fig 2(b). Moreover, a patient’s posture while sitting in the bed or chair or ambulating might not vary much as they keep an upright posture. This effect can be seen in Fig 9 showing the output from the classifier where ambiguity between sitting in bed and in the chair is shown. Although this problem was reduced using non-overlapping windows, it is the case for patients p.1, p.6, p.12, p.13, p.16, p.19, p.20, p.22 where most FPs are caused by the classifier’s inability to discriminate between these activities i.e. sitting on chair, sitting on bed and ambulating in the relatively small area next to the bed and chair. However, as expected, the use of non-overlapping windows produced most detection delays within the first processing time window. In the case of patient p.10, misclassifications occurred when the patient was lying in bed, as some readings were classified as sitting or ambulating.

The location of antenna1—focused on the chair—on the wall opposite the chair collected few sensor observations. In the case of people sitting on the chair, adequate powering of the sensor is difficult due to the possibility of the chair or the patient being not directly illuminated by this RFID reader antenna as the patient may sit leaning forward or not face the RFID antenna (antenna1) directly. Thus, both ambulation (≈ 9% of total sensor observations) and sitting on chair (≈ 22%) activities were captured with less sensor observations than lying (≈ 56%). For example, patient p.9 has only one sensor observation of the patient sitting on the chair. In the case of ambulation, sensor observations are obtained until the person walks by antenna1 when going to the door and, similarly, in the opposite direction, readings are collected when the patient is close to antenna1. Past the location of antenna1, when going to the door, none of the antennas illuminate the sensor as the patient’s body obstructs the sensor. Moreover, ambulation events can also be of short duration as the patient can access the chair located next to the bed in few short steps. This limited amount of data, especially for ambulation, manifests as poor quality information and features extracted by such sparse sensor observations do not provide adequate information about the underlying patient movements to discriminate activities and, consequently, poor activity recognition performance.

We were not able to find studies reporting both bed and chair exit results to compare with our results, and a fair comparison is indeed difficult given the often different settings, e.g. demographics of participants, sensor type and location, used by different studies. Therefore, there is difficulty in comparing our approach with previous long-term studies [4, 14, 15] as these are longitudinal trials with larger populations of hospitalized older people monitored during both on day and night time conditions that reported occurrence of falls and not alarm recognition effectiveness. For instance, the recent long term RCT from Sahota et al. [14] used bed and chair pressure sensors, their results did not report the alarming performance per apparatus but the resulting falls rate. However, the study of Capezuti et al. [4] used pressure sensors on beds in a longitudinal study to measure and report bed exit detection performance in older people where the recall of the system was of 71% and specificity 0.3% [4]. This high false alarm rate might be one reason why pressure sensors have been found to be ineffective in recent clinical trials [14, 15].

There have been other studies [54, 55] that considered only bed exits and reported recall and specificity values over 90%. However, these studies were undertaken with young and middle aged adults and that is a limitation of those studies [54, 55]. Furthermore, the empirical methods in [54, 55] were developed and tested with the same dataset, yielding optimal thresholds for heuristic measures for the particular dataset.

We compared our proposed method performance, F-score of 72%, to the results from previous studies in [36] as shown in Table 4. Comparing with the method in [36], where a weighted classifier was used to address the problem of imbalanced data, only recall was slightly lower for our method with a non-statistically significant difference of *P* = 0.58. However, precision and F-score, were higher for our method with statistically significant difference (*P* < 0.0013) than those of [36]. A possible cause is the use of a sliding window in [36] that fails to filter some false alarms, affecting the overall F-score, albeit producing slightly more true alarms.

### Acceptability study

Results from the acceptability study indicate that the sensor system appears acceptable to the hospitalized older people that participated in this pilot study. Results from the first survey denote, in general, increased confidence after the patients experienced the equipment as the overall score increased to 9.0 in the post-trial survey. Although post-trial response scores for Q6: “I am afraid the equipment will harm me” decreased after the trials for male patients and slightly improved for female patients, this indicates a concern about their safety (see Q6 in Fig 7). This is possibly due to the visible infrastructure to keep RFID antennas in place in patient’s rooms as well as the highly visible sensor prototype used in the study. The results from the second survey suggest a general acceptance of the equipment; however, lower scores regarding the patients being conscious of wearing the device (E2) were most probably due to the experimental nature of the highly visible sensor device. In terms of privacy, the overall score was high (9.1), suggesting that the patients that participated in the trial were comfortable with being monitored using the wireless sensor device in the hospital setting. Overall, the results from the two surveys indicate that: i) the participants of the trial found the sensor acceptable to use; ii) initial anxiety about the equipment can be overcome by allowing older people to experience the equipment; and iii) the development of our sensor device must aim to eventually be integrated into textile such as a vest or the hospital gown so it is less obtrusive and unnoticeable to a patient.

Overall, our results agree with previous study findings [19] and show that, despite the very visible nature of our particular sensor, wearable sensors are an acceptable technology for monitoring older people. Further, wearable sensors have the potential to overcome privacy issues raised around the use of video monitoring of older adults in previous studies [16–18]. The study in [17] recommends the avoidance of using video images for more ethical practices such as silhouette extraction; however, this technology is still not widely accepted by older adults [16, 19]. The study in [19] states that, among community-dwelling older people, cameras raised greater privacy concerns than other technologies, even when methods for extracting silhouettes were in place to preserve privacy. The study in [16] using a camera for capturing depth information (i.e. a person’s body silhouette) for assessing gait parameters in a home setting highlighted that 6 out of 15 eligible participants contacted did not want to participate in the study despite being provided an explanation and a picture of the privacy preserving silhouette images collected from the depth sensor.

In addition to our body worn sensor being able to mitigate privacy concerns, our approach also provides technical and economic advantages over video images: i) RFID hardware is increasingly deployed to support asset tracking in hospitals and our approach provides a dual use for an existing infrastructure [56]; ii) the same technology can provide solutions to monitoring not only falls risk but other conditions such as ‘wandering-off’ [31] of individual patients; iii) sensors can be capable of capturing more health-related information such as heart rate [57]; and iv) the automatic unique identification provides the ability to individualize alarms to suit the monitoring needs of individual patients.

### Limitations and future work

Our approach is not without limitations. Although the use of the non-overlapping windows method reduces some errors such as false alarms, it also bounds the delay to produce an alert for staff to intervene, as most alarms are issued within the first time window of duration 4.8 s. In addition, there will always be delays before a staff member is able to assist a patient unless the staff is stationed very near to the patient’s room, and therefore, a timely intervention also depends on the human response time. However, in the case of bed exiting, our problem formulation to consider the patient sitting on the bed as attempting to exit the bed gives extra time for staff to intervene.

The proposed intervention for falls prevention has no direct method to detect a fall. However, staff already notified of a bed or chair exit event can act upon a fall incident in the event the fall is not averted and prevent the damaging effects of a ‘long-lie’—condition where life expectancy can be severely reduced as a result of an old person staying more than one hour on the floor after a fall. In relation to the completed surveys, the unbalanced distribution of male to female population and the small size of the trialled population is a limitation to our study. Future trials must consider these population issues as well as resolution of the points raised by our surveys, i.e. highly visible sensor device and RFID infrastructure, to derive more reliable conclusions.

In terms of extending the study, given that some patients used walking aids, it would be relevant to assess ambulation without a required walking aid and alarm when an un-aided unsupervised patient attempts ambulation. This can be carried out once a bed or chair exit has been recognized, and the system can then assess whether a previously sensor tagged walking aid associated with the patient is being used. The continuous improvement of innovative techniques such as robust classifiers for activity classification using machine learning techniques, the introduction of improved motion descriptive features or the addition of extra sensors (e.g. barometers) may further improve the performance of the present pilot study and are currently being investigated. It should be highlighted that our approach is proposed for a hospital environment; applying these methods to an independent living environment, such as an older person living in their own homes, will require further development due to the greater variety of activities performed, and multiple bedroom furniture settings possible in a home environment.

Future work in the development of the intervention must address the limitation of the current study and verify improvements over the results obtained in this study. First, having all antennas ceiling-mounted can help focus on specific room locations and improve powering of the sensor as well as a simple deployment option for different rooms. Second, placing the sensor on the shoulder rather than the chest is likely to achieve better illumination from ceiling mounted antennas, avoid occlusion from objects and the patient’s body and reduce the sparsity in the sensor data, especially during ambulation. Third, in terms of sensor improvements, further research needs to improve resistance to wear-and-tear and reduce its size and visibility, as was also suggested by the results from the acceptability study, without losing performance. We have already made progress toward that end [58]. Future research must consider a trial with a larger cohort of hospitalized older people wearing a less obtrusive and more robust sensor prototype for longer periods and include scenarios such as staff interacting with patients and people visiting patients and changing the location of the chair. Ultimately, we need to evaluate the efficacy of this system to reduce falls in acute hospital settings using a RCT.

### Conclusions

We have described a movement monitoring sensor system incorporating a single RFID sensor worn by hospitalized older people that recognizes bed and chair exits and its preliminary pilot study results are promising. We have identified areas of future development to improve our system’s performance, such as placing sensors on the shoulder and RFID reader antennas on the ceiling and improving sensor performance by reducing size and energy consumption by embedded sensors. Further research may be undertaken to include a pilot study for a longer period of time with further developed infrastructure to improve our result metrics and overcome the limitations of our pilot study, and increase the activities of interest (using walking aids). Finally, the system was perceived acceptable to the patients performing the trials on all factors of the sensor acceptance model and showed, in general, an increased confidence after use.

## Supporting information

S1 Pseudocode Pseudocode of algorithms. Algorithm used in this study.
(PDF)

S1 Dataset Dataset hospital. Data corresponding to the sensor observations for hospitalized older people participating in the trials; data anonymized except for gender.
(ZIP)
