# Supplementary material for: A battery-less and wireless wearable sensor system for identifying bed and chair exits in a pilot trial in hospitalized older people
Source: PLoS One. 2017 Oct 9;12(10):e0185670. doi: 10.1371/journal.pone.0185670 (PMC5633180; doi:10.1371/journal.pone.0185670)
Supplement: S1 Pseudocode — Algorithm used in this study. (PDF) [file pone.0185670.s001.pdf]

1 Pseudo code

Algorithm 1 Activity class assignment

```
Require: Output from classifier (probability for each class) Pr_k, Number of non-overlapping windows M
for m=1 to M (M can be ∞ ) do
    y_tmp ← Σ_q (Pr_{k,q}); q ← (m − 1) × 𝔗 to m × 𝔗 .
    y_{pred,m} ← index of max_k(y_tmp)
```

Algorithm 2 Bed and chair exit recognition from assigned classes

Require: predicted output y_{pred}

```
for n=2 to N (N can be ∞ ) do
    if y_pred(n − 1) = Sit_on_bed then
        if y_pred(n) = Sit_on_chair then
        else if y_pred(n) = Lying then
        else if y_pred(n) = Ambulating then
    else if y_pred(n − 1) = Sit_on_chair then
        if y_pred(n) = Sit_on_bed then
            if previous predicted bed/chair exit occurred more than 1.75 s ago then
                BedChairExitPred(n) ← 1
        else if y_pred(n) = Lying then
            if previous predicted bed/chair exit occurred more than 1.75 s ago then
                BedChairExitPred(n) ← 1
        else if y_pred(n) = Ambulating then
            if previous predicted bed/chair exit occurred more than 1.75 s ago then
                BedChairExitPred(n) ← 1
    else if y_pred(n − 1) = Lying then
        if y_pred(n) = Sit_on_chair then
            if previous predicted bed/chair exit occurred more than 1.75 s ago then
                BedChairExitPred(n) ← 1
        else if y_pred(n) = Sit_on_bed then
            if previous predicted bed/chair exit occurred more than 1.75 s ago then
                BedChairExitPred(n) ← 1
        else if y_pred(n) = Ambulating then
            if previous predicted bed/chair exit occurred more than 1.75 s ago then
                BedChairExitPred(n) ← 1
    else if y_pred(n − 1) = Ambulating then
        if y_pred(n) = Sit_on_bed then
        else if y_pred(n) = Lying then
        else if y_pred(n) = Sit_on_chair then
```
